# Susceptibility of LoRa Communications to Intentional Electromagnetic Interference with Different Sweep Periods

**DOI:** 10.3390/s22135015

**Published:** 2022-07-02

**Authors:** Artur N. De São José, Virginie Deniau, Christophe Gransart, Thomas Vantroys, Alexandre Boé, Eric Pierre Simon

**Affiliations:** 1 COSYS-LEOST, Université Gustave Eiffel, IFSTTAR, Université de Lille, F-59650 Villeneuve d’Ascq, France; virginie.deniau@univ-eiffel.fr (V.D.); christophe.gransart@univ-eiffel.fr (C.G.); 2 Université de Lille, CNRS, USR 3380—IRCICA—Institut de Recherche sur les Composants Logiciels et Matériels pour l’Information et la Communication Avancée, F-59000 Lille, France; thomas.vantroys@univ-lille.fr (T.V.); alexandre.boe@univ-lille.fr (A.B.); eric.simon@univ-lille.fr (E.P.S.); 3 Université de Lille, CNRS, Centrale Lille, UMR 9189 CRIStAL, F-59000 Lille, France; 4 Université de Lille, CNRS, Centrale Lille, Université Polytechnique Hauts-de-France, UMR 8520—IEMN, F-59000 Lille, France

**Keywords:** LoRa, internet of things, intentional electromagnetic interference, jamming, frequency hopping

## Abstract

This work presents a detailed analysis of the susceptibility of LoRa communications in the presence of intentional jamming signals. The analysis is performed with a periodic frequency-sweeping intentional electromagnetic interference, corresponding to the most common jamming signals. Such a waveform faithfully represents the signals emitted by commercial jammers. As the sweep period of the jamming signals may vary from one such device to another, the analyses are conducted with different sweep period values, from 1 μs to 50 μs. The experimental results indicate that the impact varies significantly according to the sweep period of the jamming signal. The detailed analysis allows us to identify the jamming signals to which LoRa communications can be resilient or not as well as to identify which LoRa channels are less affected during an attack.

## 1. Introduction

Internet of things (IoT) systems are increasingly being integrated into critical infrastructures, such as smart energy grids, vehicular networks, and healthcare [1]. In particular, the railway industry has been adopting the long range (LoRa) technology to optimize the train maintenance processes and to allow predictive maintenance [2]. Indeed, many sensors are installed inside the trains to monitor the state of different equipment, but to access the sensor data, the railway staff has to go to the installation location and collect the data directly from the sensors. By associating these various sensors with a LoRa wireless communication board, the sensor data can be transmitted directly to a railway IoT platform that centralizes all the data. Sensor data can then be remotely accessed before the train enters a station or maintenance center so that only the necessary interventions are scheduled. Moreover, when the sensors ensure a safety function, such as checking that a train door is closed correctly, an alert can be transmitted to the train staff as soon as the fault is centralized on the IoT platform.

However, as LoRa communication reception can be degraded due to the train speed, LoRa-LTE gateways are installed on the trains, with one gateway per train carriage. Thus, the onboard sensors send their information in LoRa to the gateway, which can transfer it using the LTE network to the IoT platform.

Despite all the advantages, IoT systems can also be vulnerable to cyber attacks [3]. An overview of cyber attack techniques used against LoRa systems can be found in [4]. The authors describe different types of attacks such as sniffing, jamming, and wormhole in this reference. In particular, they point out the effectiveness of reactive jammers, which contain an embedded sniffer that listens to the LoRa channels and identifies the right moment to jam. Indeed, the efficacy of a jamming attack is maximized if the attacker’s and the victim’s transmitters are synchronized [5].

In [5], the authors show the potential of reactive jammers to disrupt LoRa communications, given their ability to emit fake LoRa signals synchronized with the licit ones. According to the authors, it is impossible to distinguish perfectly synchronized LoRa signals (e.g., a fake one and a licit one) in the time domain. Thus, they propose a mitigation technique based on received signal strength indication (RSSI) values. Knowing this parameter stays stable during a LoRa transmission, they try to identify the presence of jammers based on abrupt changes on the RSSI levels. An attack strategy based on insider smart jammers is described in [6]. Such an attack targets spread spectrum communication systems, e.g., LoRa, which is based on the chirp spread spectrum (CSS) modulation. In particular, the authors explore the vulnerabilities of direct-sequence spread spectrum (DSSS) systems to insider smart jammers, which have the information about one of the spreading sequences shared between network devices. To improve the system robustness, they propose the use of modern cryptographic secure pseudo-random number generators outputs to uniquely generate spreading sequences for each pair of network nodes. In a recent study, the authors in [7] propose an original anti-jamming strategy based on the imperfect orthogonality between LoRa signals with different spreading factors (SF) (a mathematical description of LoRa signals exploring their orthogonality properties can be found in [8]). Such a strategy consists in emitting friendly jamming signals which collide with non-friendly jamming signals but do not affect the demodulation of licit LoRa signals at the receiver. Other strategies, such as constant, deceptive, and random jamming attacks, are described in [9].

In [10], the authors investigate the vulnerability of LoRa communications during the join procedure, i.e., when a LoRa device tries to establish communication with a gateway for the first time. They describe a scenario where a particular field of the join request message called DevNonce is jammed, resulting in denial-of-service (DoS). In the first version of the long range wide area network (LoRaWAN) protocol, DevNonce is a RSSI-based random parameter whose value never repeats in such a way as to avoid replay attacks [4]. In this context, the emission of strong jamming signals that saturate the power measurement unit of a LoRa transceiver forces the LoRa device to use the same RSSI every time and, therefore, generate the same DevNonce, and all attempts of this node to join the network are refused. Mitigation strategies against jamming attacks designed for LoRa systems can be found in [4,5,11].

Regarding IoT applications, one of the most popular cyber-attack strategies is to create DoS to force the communicating devices to resend the messages to strain their battery and put it out of order. A means to create such a DoS, without any technical knowledge, is to use commercial jammers in the vicinity of wireless devices, such as base stations, gateways, access points, or users’ terminals. Jammers can be easily bought on the internet, they are cheap, and do not require any technical knowledge to be operated.

The commercial jammers are generally based on voltage-controlled oscillators (VCO), which allow them to jam a given frequency band by emitting a radiofrequency signal that repetitively and quickly sweeps the targeted frequency band. The effects of this type of jammer on Wi-Fi networks were already analyzed and evaluated [12,13]. However, its impacts on LoRa communications are still unknown. Indeed, in most papers about the jamming of LoRa systems, the term *jammer* refers to devices that generate fake LoRa signals, generally based on software-defined radio (SDR), and Arduino [14]. The fake LoRa signals can use the same frequency and time resources as the licit LoRa signals and prevent the gateway from receiving the messages adequately.

Since the gateway is in charge of forwarding information of several IoT devices to the servers, when a jammer is activated near a gateway, DoS can be produced on several sensors simultaneously [15]. In this work, we analyze the effects of commercial jammers on the gateway reception of the LoRa uplink signals, which are the signals sent by the sensors to the gateways. Moreover, in general, these jammers are designed to be able to jam several communication systems (Wi-Fi, 2G, 3G, 4G, etc.) simultaneously. Even if they do not specifically target the LoRa communications, they can still degrade such a communication system.

In this paper, we describe an experimental methodology to analyze the electromagnetic (EM) susceptibility of LoRa communication links in the presence of periodic frequency-sweeping intentional electromagnetic interference (IEMI). This particular waveform faithfully represents the signals emitted by commercial jammers. Our methodology can be used to identify which LoRa channels are less affected in these circumstances. Therefore, it has the potential to be used together with intrusion detection systems (IDS) and frequency hopping (FH) to enhance the robustness of LoRa devices. Indeed, some authors have recently proposed alternative channel access strategies for LoRa networks, given the limitations of the traditional ALOHA technique [16,17,18].

Our interest in the railway domain is explained by a research project conducted with the SNCF, the French national railway company, which considers that this kind of jammers presents a real threat because everyone can use them. However, the analyses exposed in this paper are valid for any other application of LoRa networks.

To the best of our knowledge, the analyses conducted in this paper were not yet explored by other researchers. In most papers about LoRa communications, the terms *interference* and *jammers* usually refer to other LoRa devices. When such devices are licit LoRa users, the goal is to design algorithms that allow a gateway to precisely identify each LoRa user in a scenario where multiple LoRa signals are simultaneously received [19]. On the other hand, some authors explore the use of illicit LoRa transmitters, which try to cause collisions between licit and illicit LoRa signals at the gateway input terminal. This operation results in the loss of licit LoRa signals [14]. In our bibliographical review, we did not find any paper exploring the behavior of LoRa communications in the presence of periodic frequency-sweeping IEMI, which represent the jammer signals emitted by the most widespread cheap commercial jammers [12]. Knowing that anyone can buy such a jammer on the Internet and use it without any specific knowledge, this jamming signal is considered a significant threat to railway applications and has to be studied.

The main contributions of this work can be summarized as follows:

- A spectral analysis that shows how a LoRa receiver processes a periodic frequency-sweeping IEMI with different sweep periods;
- A detailed analysis which shows that the impact of the jamming signal depends on its sweeping period as well as on the attacked frequency band;
- An experimental study based on error indicators and different transmitting frequencies showing that certain jamming signals do not affect all LoRa channels in the same way.

This paper is organized as follows. In Section 2, we describe the behavior of LoRa signals in the frequency–time domain. In Section 3, we present the periodic frequency-sweeping jamming signal, and then we explain how a typical radio receiver processes it. In Section 4, we present the test bench, and we formalize the research methodology. Finally, we present the main results in Section 5 and we summarize the main findings of this investigation in Section 6.

## 2. LoRa Communications

The term LoRa communications refers to the physical layer of the LoRaWAN protocol. LoRa is a patented technology [20] which belongs to the Semtech company, while LoRaWAN is an open protocol managed by LoRa Alliance. In this work, we focus on the physical layer only. So, in this section, we describe the LoRa frame structure, as described by Seller and Sornin [20], as well as the time–frequency behavior of each symbol.

**Figure 1:**
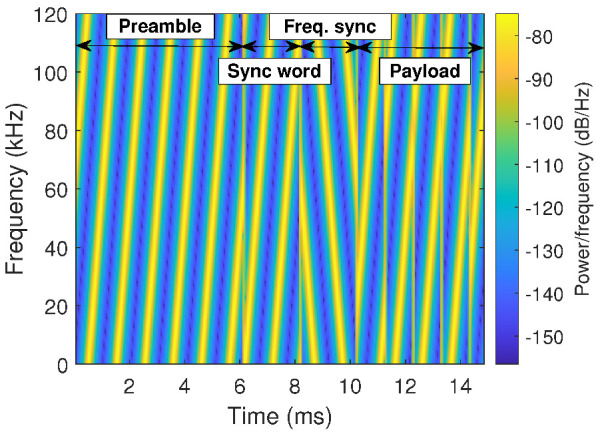
Spectrogram illustrating a baseband LoRa frame structure (SF=7, $T_{\mathrm{sym}}=1\;ms$, B=125 kHz). The three first elements (preamble, frame sync and frequency sync) are used for the synchronization process, and the last one (payload) contains the sensor data. Here, we only show four payload symbols to facilitate the visualization.

The structure of a LoRa frame can be partially seen on the baseband time–frequency plot shown in Figure 1. This spectrogram reveals that a LoRa frame consists of up-chirps and down-chirps, i.e., frequency-sweeping signals with increasing and decreasing frequencies, respectively. Each of them carries the information of one symbol and has a time duration of $T_{\mathrm{sym}}=2^{\mathrm{SF}}/B$ seconds, where SF is a parameter called spreading factor (possible values: 7,8,9,10,11,12) and B is the bandwidth (usually, B=125 kHz or B=250 kHz).

**Figure 2:**
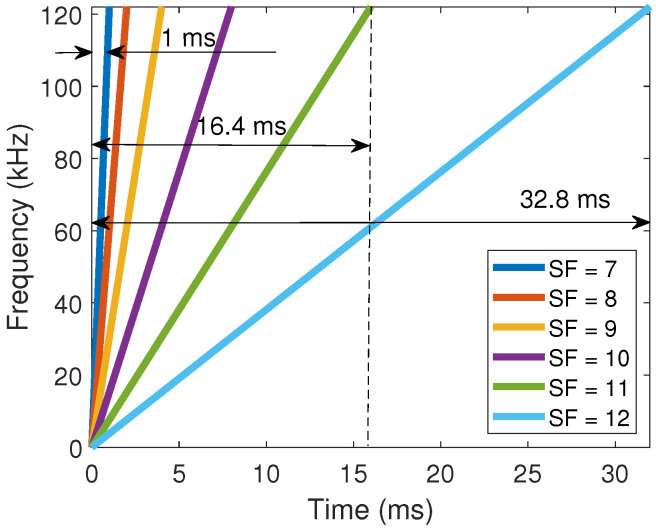
Time–frequency plot of six raw LoRa upchirps, each one with a given SF.

To better understand the impact of the SF parameter, let us analyze the six raw LoRa upchirps illustrated in Figure 2. They represent unmodulated LoRa signals transmitted during $T_{\mathrm{sym}}$ seconds. Each curve describes the time–frequency behavior of the LoRa signal with a given SF. In this figure, the SF parameter was progressively increased from 7 up to 12, covering thus all the available SF values.

The graphs in Figure 2 reveal a linear behavior between frequency and time. If we want to use one of these waveforms to send one symbol, we first define the following set of integer numbers: $m=\{0,1,...,2^{\mathrm{SF}}-1\}$. Then, we define a SF, we select the symbol index m from this set, and we apply a shift of magnitude m/B to the corresponding curve in Figure 2. Although the symbol is spread in time, the relevant information is the time instant where the instantaneous frequency switches from its maximum to its minimum value. This time instant is unique for each symbol since it is related to the shift m/B. Therefore, the presence of interference and noise in other time instants has a low impact. This makes the CSS a relatively robust technique compared to other modulation schemes.

The different slopes shown in Figure 2 refer to the choice of the SF parameter. Lower SF values indicate short symbol times and, therefore, higher throughputs, reducing the communication reliability and therefore range. On the other hand, higher SF represents more reliable communication links with a longer range, but at the cost of throughput. Therefore, the choice of this parameter is a trade-off that depends on the application.

To better understand the LoRa frame structure, let us return to Figure 1. In this figure, the first three fields are related to the synchronization process, where the receiver identifies the beginning of a frame. If this stage is not successful, the receiver discards the received signal. Our test methodology considers synchronization problems when analyzing the jamming effects over the LoRa communications. Indeed, if the interference affects the integrity of this specific part of the frame, the entire LoRa packet is lost (even if the payload is not corrupted). More details about these particular fields of the LoRa frame and the synchronization process can be found in [21]. The authors propose low-complexity LoRa detection algorithms capable of dealing with timing and frequency synchronization issues.

As a last remark about the LoRa frame structure, it is worthwhile to mention the presence of two cyclic redundancy check (CRC) bit sequences: one inside the header (optional field positioned between the synchronization symbols and the payload) and another one at the end of the payload. It allows the receiver to detect errors in the received frame. Furthermore, a forward error correction (FEC) is added to the data, implemented by encoding redundancy bits. In this context, the coding rate (CR) is defined as the proportion of useful bits within a block of transmitted data. For example, CR=4/5 means that, within a block of 5 transmitted bits, 4 of them are useful bits and 1 is an error-correction bit. In LoRa communications, typical CR values are 4/5, 4/6, 4/7, 4/8 [22], where CR=4/5 and CR=4/8 respectively represent 1/5=20% and 4/8=50% of error-correction bits. Consequently, they represent the worst- and the best-case scenarios of protection against interference and noise, respectively. However, the price for a larger amount of error-correction bits is a reduction of the network throughput. In this work, we adopt CR=4/5, which is a worst-case scenario in terms of resilience to interference.

## 3. Jamming Signal

The jamming signal considered for this investigation is a periodic frequency-sweeping sine wave. This signal corresponds to an actual jamming signal emitted by a commercial jammer bought on the Internet. We previously measured and analyzed jamming signals emitted by commercial jammers, and we noticed that some of them could cover a 140 MHz frequency band, including the LoRa, 2G, and 3G frequency bands. We then focused our study on this type of jamming signal.

**Figure 3:**
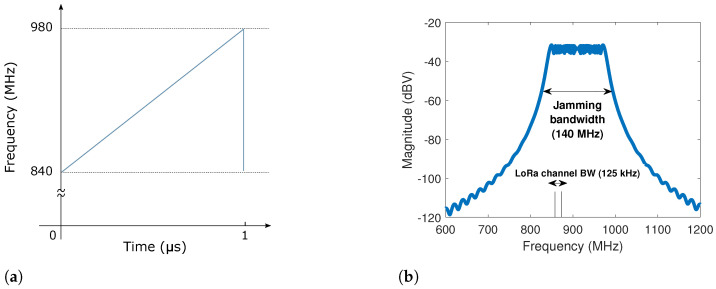
(**a**) Time–frequency representation and (**b**) magnitude spectrum of one single jamming signal cycle. This jamming signal has a sweep period of 1 μs, a bandwidth of 140 MHz (840 MHz to 980 MHz) and an amplitude of 250 mV. We also highlight how narrow the LoRa channel bandwidth is (125 kHz) in this context.

To explain the behavior of this signal, let us start with the elementary jamming cycle. During a relatively short period (also known as *sweep period*), the sine wave covers a wide frequency band, as illustrated in Figure 3a. This time–frequency plot highlights the linear relationship between frequency and time. Such behavior can be mathematically expressed as follows [12]:

(1) $$i\left(t\right)=A\cos\left[2\pi \left(\frac{f_{2}-f_{1}}{2T_{\mathrm{jam}}}t+f_{1}\right)t\right],$$

where A is the amplitude, $T_{\mathrm{jam}}$ is the sweep period, $f_{1}$ and $f_{2}$ are the lowest and highest frequencies which define the signal bandwidth and t is the time, which is defined within the interval $0<t<T_{\mathrm{jam}}$. The resulting magnitude spectrum is continuous and approximately flat, as shown in Figure 3b.

**Figure 4:**
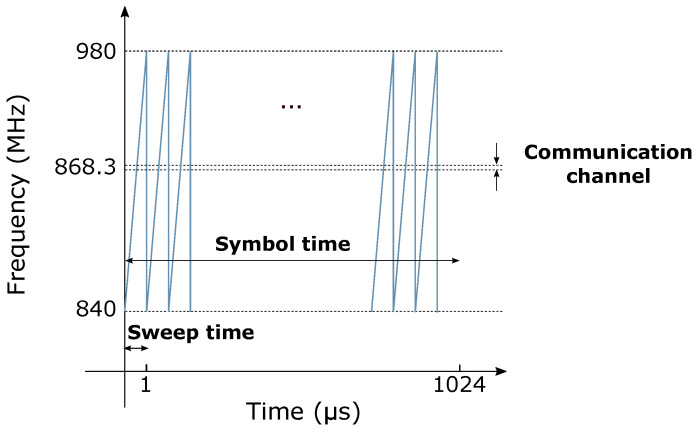
Time–frequency representation of a 1 μs jamming signal. This graph shows that, during $T_{\mathrm{sym}}=1.024\;ms$, a SF=7 LoRa receiver is subject to 1024 jamming signal cycles.

The signal illustrated in Figure 3a,b represents a single cycle of the signal generated continuously by the jammer. However, it does not represent the one seen by a LoRa receiver. Indeed, the jamming signal is distorted by the processing carried by the LoRa receiver. The main reason is that the IEMI sweep and repetition period is fast compared to the LoRa symbol time and to the integration time window. Compared to LoRa symbols, whose duration ranges between 1.024 ms for SF=7 and 32.768 ms for SF=12 when B=125 kHz (respectively, 0.512 ms and 16.384 ms when B=250 kHz), jamming signal cycles are fast, with sweep periods typically ranging from 1 μs to 50 μs. As a result, when a LoRa demodulator tries to decode the received symbol, it also processes, during $T_{\mathrm{sym}}$ seconds, an undesirable signal consisting of several jamming cycles. For example, if we consider the case where $T_{\mathrm{jam}}=1\;\mu s$, B=125 kHz and SF=7 ($T_{\mathrm{sym}}=1.024\;ms$), then the time window used by a LoRa receiver to decode one symbol corresponds to 1024 jamming signal cycles, as illustrated in Figure 4.

Figure 4 is a time–frequency plot that illustrates how the jamming signal is perceived by a SF=7 LoRa receiver. We can see that, during $T_{\mathrm{sym}}=1.024\;ms$, the LoRa receiver is exposed to 1024 jamming cycles. Furthermore, since the LoRa bandwidth is very narrow compared to the IEMI bandwidth, the jamming signal is most of the time outside the communication channel.

**Figure 5:**
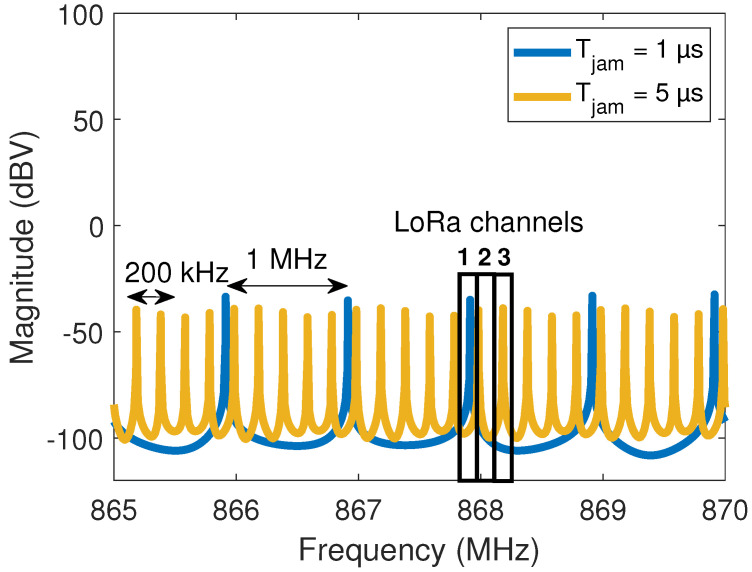
Jamming signal spectra ($T_{\mathrm{jam}}=1\;\mu s$, $T_{\mathrm{jam}}=5\;\mu s$ and amplitude equal to 250 mV) seen through a 1.024 ms window of a LoRa demodulator (SF=7). The spectrum spans from 840 MHz to 980 MHz, but in this figure, we only show the range from 865 MHz to 870 MHz.

Therefore, from the point of view of a LoRa receiver, the IEMI behaves like a finite-length periodic signal. As a consequence, the IEMI spectrum shown in Figure 3b is no longer homogeneously distributed along all the LoRa channels, as we can see in Figure 5. The graphs shown in this figure correspond to the cases where $T_{\mathrm{jam}}=1\;\mu s$ and $T_{\mathrm{jam}}=5\;\mu s$. They reveal that the distance between two consecutive jamming components is $1/T_{\mathrm{jam}}$.

The spectra shown in Figure 5 are obtained by applying a fast Fourier transform (FFT) on the jamming signal over a 1.024 ms time window. This 1.024 ms duration corresponds to the duration of one LoRa symbol with a SF=7. We notice that the spaces between the main frequency components are 1 MHz for a 1 μs jamming signal cycle and 200 kHz for a 5 μs jamming signal cycle. The energy of slower jamming signals is more densely spread in the frequency domain. This figure also reveals that the impact of the jamming signal can depend on the LoRa channel frequency due to the jamming signal not being uniformly distributed over all the frequencies. To illustrate this aspect, we highlight, in Figure 5, the location of three hypothetical LoRa channels. Channel 1 is affected by both interfering signals, Channel 2 is interference free, and Channel 3 is only affected by the 5 μs IEMI. So, if an IDS would identify a 1 μs IEMI when a LoRa transmission occurs in Channel 1, then a FH algorithm can be triggered to change the transmission either to Channel 2 or to Channel 3. On the other hand, if a 5 μs IEMI is identified, then the only free channel would be Channel 2. This example shows that the faster the jamming signal is, the fewer are the interference-free channels.

The frequency intermittency of the received jamming signal is an important characteristic, which is not only due to the definition of the jamming signal but also to the time window and frequency resolution of the LoRa processing receiver. Moreover, the jamming signal is sometimes inside and sometimes outside the LoRa channel boundaries. This happens because the 140 MHz IEMI bandwidth is much larger than the 125 kHz LoRa bandwidth (see Figure 3a). The amount of time spent inside and outside the channel depends on the jamming sweep period, the interference bandwidth, and the LoRa signal bandwidth. The consequence is a fluctuation of the instantaneous IEMI power levels seen by the LoRa receiver—more details about this problem can be found in [23]. These power fluctuations, in turn, impose a challenge to the signal-to-interference ratio (SIR) calculation and control in a lab environment. In Section 4.2, we detail an appropriate methodology for such tasks.

## 4. Methodology

In this section, we describe our experimental methodology in terms of the test bench, SIR calculation, and error indicators. We firstly detail the equipment used and how they are interconnected. Then, we describe how we calculate the jamming signal power in order to estimate and control the SIR. Finally, we introduce the error indicators used to characterize the LoRa communications in the presence of IEMI.

### 4.1. Test Bench

The main objective of the test bench is to analyze the effects of periodic frequency-sweeping IEMI on the LoRa communications without taking into account other interference sources. In this context, it is not our goal to analyze neither the impact of multipath nor the impact of any misalignment between the LoRa transmitting and receiving antennas. So, the test setup must ensure that all these EM effects are avoided. Finally, with our test bench, we must be able to simulate a scenario, where the attacker can be located at different distances or even move and, therefore, the IEMI power levels at the LoRa gateway can change along the time.

The proposed test bench was inspired by other similar test benches, such as those described in [24,25]. To observe the impacts of IEMI on a LoRa communication link without the influences of other radiofrequency signals present in the test environment, we replaced the LoRa transmitting and receiving antennas as well as the jammer transmitting antennas by shielded coaxial cables. This allows us to isolate the signals of interest from all others. Alternatively, we could have moved the test setup to an anechoic chamber. However, we could still have problems with the repeatability of the results. One of the main reasons is that it is challenging to place all these antennas in the same positions every time we run the test. In an electromagnetic compatibility (EMC) laboratory, where the anechoic chamber is shared among different users, it may be needed to remove the test setup and redo all the connections on another day. However, it is very probable that the alignment between the jammer transmitting antennas and the LoRa transmitting and receiving antennas will not be identical to those of the previous measurement campaign. Therefore, the EM coupling between the devices would not always be the same, which will in turn affect the repeatability of the test results. Additionally, we are neither investigating fading nor multipath, so we can consider the propagation conditions of coaxial cables.

**Figure 6:**
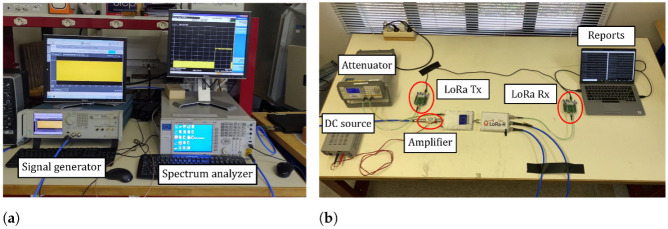
Pictures of the test setup. The main equipment are (**a**) a signal generator and a signal analyzer and (**b**) LoRa transceivers, splitters, an amplifier fed by a direct current source, a variable attenuator and a laptop to monitor the communication.

**Figure 7:**
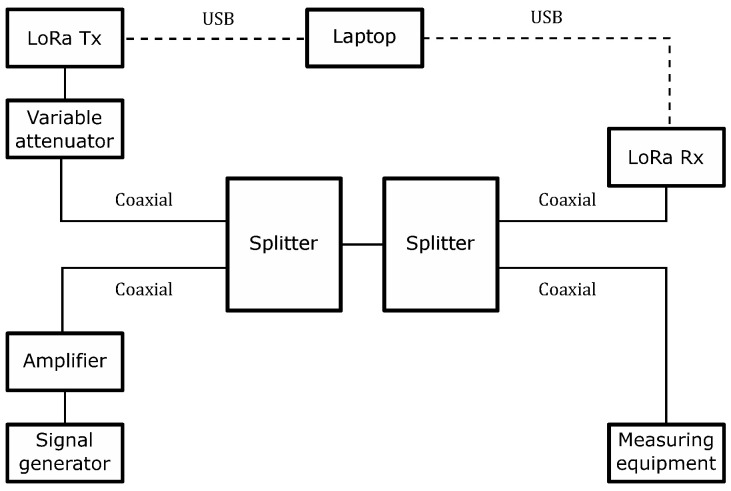
A block diagram representation of the test bench.

Figure 6 shows a picture of the test setup implemented at the Electromagnetic Compatibility Laboratory of the Gustave Eiffel University (Villeneuve d’Ascq, France), while Figure 7 contains a block diagram summarizing the equipment and connections used during the tests. As we can see in Figure 7, we combine a LoRa uplink signal with a jamming signal synthesized with an arbitrary signal generator. Two splitters are used to combine these two signals and make the resulting waveform samples simultaneously available for both the LoRa receiver and the measuring equipment. We also use a variable attenuator to control and vary the SIR. The control of the SIR levels is detailed in Section 4.2.

Several files were created to generate the jamming signal, consisting of signal samples in the time domain. Equation (1) was implemented in MATLAB with a fixed amplitude and bandwidth and a variable sweep period. All files are loaded in the arbitrary signal generator, where they are periodically generated. The jamming signal amplitude is 250 mV at the signal generator output, while its frequency band is 840 MHz to 980 MHz. A 12 V amplifier is connected at the signal generator output to boost the jamming signal power. During the experiments, different sweep periods were evaluated in the 1 μs to 50 μs range.

We used two identical SX1272 transceivers, each one containing the implementation of the LoRa communication in the 868 MHz frequency band (EU863–870 [26]) with a particular set of parameters. This type of equipment allowed us to operate at the physical layer only, without all processes related to the upper layers of the LoRaWAN protocol. We connected both transceivers to a laptop with a pair of USB cables. These connections allowed us to burn the embedded software for the transceivers and read information about the data traffic. During the execution of the tests, timestamps are added to each data sent. The payload content is displayed, and, more specifically, the values of two counters (one present at the beginning of the payload and the other at the end) are highlighted. Simultaneously, we obtained the receiver report delivering the same type of information. The difference between the two reports (i.e., the reports provided by the transmitter and the report provided by the receiver) should be null when no interference occurs, indicating that 100% of the data sent are correctly received. Still, the reports can differ in the presence of interference since not all the transmitted bytes are received. It was possible to stop the transmissions anytime. Furthermore, a specific Python routine was designed to obtain a summarized report of errors. This type of report contains some error indicators, whose details are given in Section 4.3.

Our idea is to demonstrate that the LoRa system can change its operating frequency when a jamming signal with the characteristics described in Section 3 is detected. The new frequency must be located in the “dead area” between two spectral components of the jamming signal (see Figure 5).

### 4.2. Signal-to-Interference Ratio

As exposed in Section 3, the power measurement needed for the SIR estimation is not straightforward in this investigation. A measurement method based on the instantaneous power levels will not lead to consistent results, due to the fast dynamics of the jamming signal, compared to the LoRa symbol time. In this context, our first proposal to assess the SIR value was a methodology based on a time–domain analysis [23]. This proposal consists in separately measuring signal and interference within a time interval of $T_{\mathrm{sym}}$, using an oscilloscope, and then post-processing the signals in MATLAB. The post-processing consists in a filtering stage, followed by an average power calculation. However, this method is time consuming because it demands post-processing in a computational environment.

So, another approach was developed. The measuring instrument that we used (an Agilent PXA N9030A signal analyzer) offers a functioning mode called *channel power*. After some parameters are defined, among which are the resolution bandwidth, the integration bandwidth, and the sweep period, the equipment starts to provide the power measurements. In particular, the integration bandwidth plays the role of a typical radio receiver band-pass filter, while the sweep period is, in this investigation, equivalent to the demodulation window (1.024 ms for SF=7).

An essential aspect of our SIR calculation methodology is the choice of an appropriate integration bandwidth value. When it comes to the LoRa power measurement, we naturally adopt the communication channel bandwidth equal to 125 kHz. However, applying the same parameter to the jamming power measurement may result in a biased and very variable measurement result of the EM attack intensity due to the non-uniform distribution of the jamming signal over the different LoRa channels. To deal with this situation, we consider an integration bandwidth equal to 10 MHz for the jamming power measurements, which is the entire frequency band that is allowed for LoRa communications in the EU863–870 [26]. This sufficiently large frequency band ensures that the power measurements are stable, regardless of the LoRa channel.

During the measurements, it was verified that both SIR estimation techniques (the one described above and the oscilloscope-based one [23]) present similar results, with a maximum difference of 2dB. Since these two methods have similar accuracy, we adopted the fastest procedure, i.e., the one based on the signal analyzer measurements.

### 4.3. Error Indicators

To analyze the susceptibility of LoRa communications face to jamming signals, we have to determine communication error or quality criteria to control with and without interference. The frame sent by the LoRa transmitter contains a 64-byte payload, including two counters which store the packet index. These two counters are useful for a fast diagnostic of the uplink. They allow us to control whether certain messages are not received by comparing the counter values simultaneously at the transmitter and receiver sides. In this context, three situations can be noticed: (i) the counter values in the receiver coincide with those in the transmitter, (ii) the counter values in the receiver do not coincide with those in the transmitter, presenting inconsistent values, and (iii) the difference between two consecutive counter values in the receiver is not unitary (e.g., in the following sequence of counter values 1,2,3,6,7…, the “jump” between 3 and 6 indicates the loss of 2 packets). The first case is the normal situation, while the other two situations come from interference effects. The rest of the message is a known and fixed sequence (0xDE, 0xAD, 0xBE, 0xEF, 0x01, 0x02, …).

In addition to the message counters, a Python routine was used to obtain supplementary indicators about the UL signal reception quality. This routine reports numerical values for the three following variables, all of them given in percentage relative to the number of bytes sent:

- Received and corrupted bytes (%): percentage of error when comparing sent and received bytes;
- Timeout (%): this type of error occurs if no frame is detected during a pre-established time interval;
- Rx error (%): percentage of discarded frames due to a synchronization issue.

The tests carried out on the LoRa communications face to the jamming signals were repeated in applying different SIR thanks to the variable attenuator included in the test bench. To define the critical SIR, we consider the first error occurrence, which can correspond to any error among the four ones described above.

## 5. Results

The results are exposed in two steps. In the first step, three different indicators, defined in the previous section, are analyzed for two different LoRa transmitting frequencies (868.0 MHz and 868.3 MHz) in the presence of jamming signals with different sweep periods and different SIR levels. In the second step, the critical SIR, corresponding to the first error occurrence, is compared for six different LoRa channel frequencies.

### 5.1. Evaluating Error Indicators at 868.0 MHz and 868.3 MHz

**Figure 8:**
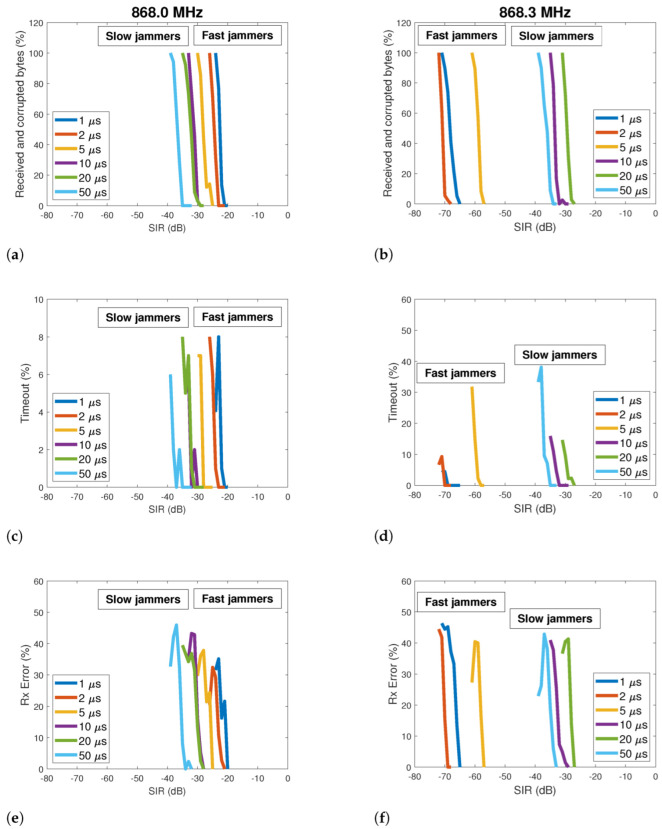
Three different indicators related to the reliability of a LoRa communication link (SF=7, CR=4/5, B=125 kHz) under jamming attack. Six different jamming signals were generated, each of them with a particular sweep period. LoRa transmission frequency: 868.0 MHz (**a**,**c**,**e**) and 868.3 MHz (**b**,**d**,**f**).

The robustness of a LoRa communication system facing jamming attacks can be expressed through the three indicators defined in Section 4.3. Figure 8 shows how these indicators vary according to the SIR, considering six different jamming sweep periods (1,2,5,10,20 and 50 μs) and two LoRa transmitting frequencies: 868.0 MHz and 868.3 MHz. To facilitate the interpretation of the results, we grouped the jamming signals into two categories: fast ($T_{\mathrm{jam}}\leq 5\;\mu s$) and slow ($T_{\mathrm{jam}}>5\;\mu s$).

All curves in Figure 8 show a decaying behavior of the error indicators when the SIR increases. The errors at the reception normally decrease when the intensity of the attack is reduced. More specifically, curves shown in Figure 8a,c,e are concentrated in a SIR range between −20 and −40dB. Although the curves are very close to each other, we can see that the slower jammers (10,20 and 50 μs) are associated with the lower SIR values. This indicates that the LoRa communication system is more robust to slower jammers when the transmission frequency is 868.0 MHz. On the other hand, the faster jammers (1,2 and 5 μs) are closer to the positive axis, indicating that the communication system is more susceptible to these sweep periods.

In the second experiment, the LoRa transmission channel is set to 868.3 MHz, and a different behavior is noted, as we can see in Figure 8b,d,f. Two significant differences with the previous results can be mentioned. While the 868.0 MHz LoRa system is more susceptible to fast jammers, the 868.3 MHz system is more sensitive to slow jammers. A comparison in Figure 8a–f reveals that the curves have their locations inverted in the SIR domain, i.e., now the slow jammer curves are closer to the positive axis. A second significant difference is a clear separation between the fast and slow jammers curves with the 868.3 MHz operating frequency. These curves now occupy different SIR areas: the slow jammers lie under the range between −20 and −40dB (similar to all 868.0 MHz curves), while the fast jammers occupy the range between −57 and −72dB. It means that the LoRa system operating at 868.3 MHz can be robust to fast jammers once this type of EMI is associated with very low SIR levels.

### 5.2. EM Susceptibility as a Function of the Channel

To understand why the EM susceptibility of the LoRa system changes so abruptly with a relatively small transmitting frequency shift, we conducted a second measurement campaign. We now consider the following transmitting frequencies: 868.0,868.1,868.2,868.3,868.4 and 868.5 MHz. Six independent transmissions at different moments were done. In each case, low EMI levels were initially used and progressively increased. Finally, the critical SIR levels were recorded, i.e., the maximum SIR for which a communication error occurs.

**Figure 9:**
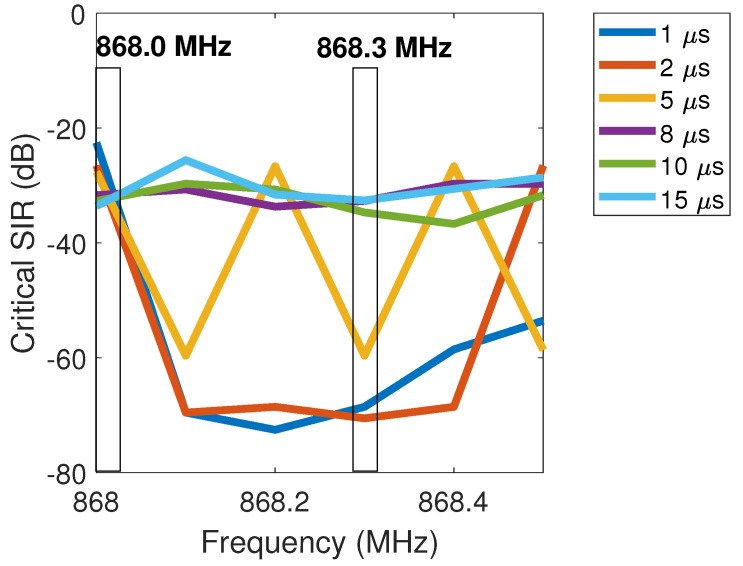
Critical SIR levels as a function of the LoRa frequency channel. Jamming signal parameters: 840 MHz to 980 MHz, variable sweep period. LoRa parameters: SF = 7, CR = 4/5, B = 125 kHz. The two channel frequencies which were previously evaluated through the different error indicators are highlighted.

Figure 9 shows the critical SIR levels according to the LoRa channel frequency. The LoRa communication was submitted to different EM attacks, each of them with a particular sweep period. When $T_{\mathrm{jam}}=1\;\mu s$, the critical SIR abruptly decreases with small shifts from 868.0 MHz. If we consider the channel frequencies generally employed according to [26], which are (868.1,868.3 and 868.5 MHz), we see that 868.1 and 868.3 MHz present lower critical SIR levels (approximately −70dB) when compared to 868.5 MHz (approximately −60dB) in the presence of the 1 μs jammer. Therefore, if a monitoring system can detect this type of attack, a simple transmitting frequency shift can significantly increase the robustness of the communication system.

When $T_{\mathrm{jam}}=2\;\mu s$, a considerable performance gain of 40dB is obtained by changing the transmitting frequency from 868.5 MHz to 868.3 MHz or 868.1 MHz. On the other hand, the 5 μs jammer presents an oscillating behavior. Fortunately, the planned channel frequencies, according to [26] lie at the minimum points of these curves, indicating that a LoRa system operating in the EU863–870 band is robust to 5 μs jammers.

The last group encompasses the slower jammers (8 μs to 15 μs). We can see that the critical SIR is hardly sensitive to the transmitting frequency shifts. Performance gains of less than 5dB can be obtained in this case, which suggests that the proposed channel selection method is more efficient when the jamming sweep period is fast (≤5 μs).

More generally, the behavior of all curves in Figure 9 can be explained by considering the frequency distribution of the jamming signal in Figure 5, seen through a 1.024 ms demodulation window. The communication is damaged if one of the jamming frequency components coincides with the employed LoRa channel. This is the case of all maximum points in Figure 9. The distance between these peaks is, in turn, $1/T_{\mathrm{jam}}$.

### 5.3. Channel Bandwidth Consideration

To understand the role played by the LoRa channel bandwidth in our methodology, let us consider two curves among the six ones illustrated in Figure 9: the red one (2 μs) and the yellow one (5 μs). Additionally, let us consider the following LoRa commercial bandwidths: 125 kHz, 250 kHz and 500 kHz. By following the red and yellow curves in Figure 9, we can see that it is strategical to place the LoRa channel in between two peaks, i.e., in a area where the critical SIR is minimal and therefore the LoRa communication is more robust to IEMI.

For the case where $T_{\mathrm{jam}}=2\;\mu s$, this area encompasses a relatively large frequency range between 868.1 MHz and 868.4 MHz. In that case, a LoRa channel centered, for example, at 868.3 MHz with a bandwidth of 125 kHz or 250 kHz would almost entirely lay down over the minimum SIR area, meaning that these are interference-free channels. However, a LoRa channel centered at the same frequency but with a bandwidth of 500 kHz would partially occupy the minimum SIR area and partially the maximum SIR area. So, in that case, only the 125 kHz or 250 kHz LoRa bandwidths are compatible with the proposed FH scheme.

If we perform the same analysis for the case where $T_{\mathrm{jam}}=5\;\mu s$, we realize that only the 125 kHz LoRa bandwidth is compatible with the proposed FH scheme. This can be understood by following the corresponding yellow curve in Figure 9. The distance between two consecutive peaks is 200 kHz. This “dead area” is narrower than two LoRa bandwidths, which are 250 kHz and 500 kHz. So, the only LoRa bandwidth that “fits” into this “dead area” is 125 kHz.

The analysis described above indicates that the 125 kHz LoRa bandwidth is the one that better suits the proposed FH scheme. So, for maximum efficiency of this methodology, it is necessary to reduce the LoRa bandwidth to 125 kHz as soon as the IEMI is detected. The drawback, in this case, is a reduction of the maximum throughput at the cost of resilience to periodic frequency-sweeping IEMI.

## 6. Conclusions

We performed an EM susceptibility analysis of a LoRa communication system facing jamming attacks. Our investigation was conducted with LoRa communication signals configured with a spreading factor of 7, which is optimal in terms of energy consumption [27]. The study is based on realistic interfering waveforms emitted by cheap commercial jammers found on the market. Contrary to the jamming signals emitted by fake LoRa nodes, the interfering signals studied here have time cycles much faster than the LoRa signals. As a result, the IEMI spectrum seen by a LoRa receiver is not homogeneously distributed over all the LoRa channels.

Experimental results demonstrate that fast jamming signals ($T_{\mathrm{jam}}\leq 5\;\mu s$) have not the same impact on all LoRa channels. For example, we noticed that the critical SIR of a LoRa communication under the presence of a 5 μs IEMI could decrease 40 dB if we change the channel frequency from 868.2 MHz to 868.3 MHz. However, slow jamming signals ($T_{\mathrm{jam}}>5\;\mu s$) disturb all LoRa channels with a relatively similar intensity.

These results suggest that if the LoRa receiver can identify the presence of fast jammers, then a channel frequency shift can significantly increase the communication system’s robustness. This goal can be achieved with an IDS devoted to the jamming signal behaviors described here, serving as an input to a FH stage (the former usually is present in LoRa receivers). Future studies can include the extension of our analyses to other LoRa spreading factors.

## Data Availability

Not applicable.

## Abbreviations

The following abbreviations are used in this manuscript:

CR	Coding Rate
CRC	Cyclic Redundancy Check
CSS	Chirp Spread Spectrum
DoS	Denial-of-Service
DSSS	Direct-Sequence Spread Spectrum
EM	Electromagnetic
EMC	Electromagnetic Compatibility
FEC	Forward Error Correction
FFT	Fast Fourier Transform
FH	Frequency Hopping
IDS	Intrusion Detection Systems
IEMI	Intentional Electromagnetic Interference
IoT	Internet of Things
LoRa	Long Range
LoRaWAN	Long Range Wide Area Network
RSSI	Received Signal Strength Indication
SDR	Software-Defined Radio
SIR	Signal-to-Interference Ratio
SF	Spreading Factor
VCO	Voltage-Controlled Oscillator
